# Differences between neonatal units with high and low rates of breast milk feeding for very preterm babies at discharge: a qualitative study of staff experiences

**DOI:** 10.1186/s12884-024-07039-0

**Published:** 2024-12-26

**Authors:** Jenny McLeish, Annie Aloysius, Chris Gale, Maria Quigley, Jennifer J. Kurinczuk, Fiona Alderdice

**Affiliations:** 1 NIHR Policy Research Unit in Maternal and Neonatal Health and Care, National Perinatal Epidemiology Unit, Nuffield Department of Public Health, University of Oxford, Old Road Campus, Headington, Oxford, OX3 7LF UK; 2 Department of Neonatology, Imperial College Healthcare NHS Trust, London, UK; 3 Neonatal Medicine, School of Public Health, Faculty of Medicine, Imperial College London, Chelsea and Westminster Hospital Campus, London, SW10 9NH UK

**Keywords:** Neonatal unit, Very preterm, Breastfeeding, Expressing, Qualitative, Staff experiences, Barriers, Facilitators, Family integrated care

## Abstract

**Background:**

Breast milk has significant benefits for preterm babies, but ‘very preterm’ babies are unable to feed directly from the breast at birth. Their mothers have to initiate and sustain lactation through expressing milk for tube feeding until their babies are developmentally ready to feed orally. There are wide disparities between neonatal units in England in rates of breast milk feeding at discharge. This study explored health professionals’ experiences of barriers and facilitators to their role in supporting breast milk feeding and breastfeeding for very preterm babies.

**Methods:**

12 health professionals were interviewed, from four neonatal units in England with high or low rates of breast milk feeding at discharge. Interviews were analysed using comparative thematic analysis.

**Results:**

Five themes were developed: ‘The role of the infant feeding specialist’, ‘Achieving a whole team approach to breast milk feeding’, ‘Supporting initiation of breastfeeding’ ‘Supporting long-term expressing’, ‘Supporting the transition to breastfeeding’. There were notable differences between neonatal units in the time allocated to specialist feeding support, the team’s sense of collective responsibility for supporting feeding, leadership, the use of external standards as levers for change, and training for the multi-disciplinary team. The feeding challenges faced by mothers of very preterm babies could be made worse where there was no joined-up working between neonatal and postnatal staff; inadequate facilities for mothers to stay with their babies; and when opportunities were missed to give information about the importance of early initiation of expressing and to support mothers’ confidence during the transition to direct breastfeeding.

**Conclusions:**

Effective support can be influenced by having a supernumerary post dedicated to infant feeding; strong leadership that champions breast milk feeding and breastfeeding within Family Integrated Care; maintaining accountability by using existing quality improvement tools and accredited standards for neonatal units; and training for the whole multi-disciplinary team that encourages and enables every member of staff to take an appropriate share of responsibility for consistently informing and assisting mothers with expressing and breastfeeding. Joined-up working between staff on antenatal and postnatal wards and neonatal units is important to enable integrated feeding support for the mother-baby dyad.

## Background

Breast milk, which is optimal nutrition for all babies, has significant additional benefits for preterm babies, such as lower rates of infections and necrotising enterocolitis, faster development of the gastrointestinal tract, and improved neurodevelopmental outcomes [1–3]. Providing breast milk for a baby in a neonatal unit may also have psychological benefits for the mother [4]. Establishing and sustaining lactation is particularly challenging for mothers who have had a ‘very preterm’ birth (before 32 completed weeks of gestation) [4, 5]. Their production of breast milk may be delayed, and they need to maintain lactation by expressing milk for tube feeding during a prolonged period until the baby is developmentally ready to feed directly from the breast [4, 6]. Production of breast milk is optimised by stimulating lactation as soon as possible after birth, and maintaining it by expressing multiple times every day, with frequency and duration of pumping being associated with higher volumes of milk production [7–9].

Establishing direct breastfeeding is also challenging for mothers of very preterm babies on neonatal units [4], but this is important as exclusive direct breastfeeding is associated with longer term breastfeeding after leaving the neonatal unit [10, 11]. Transition to direct breastfeeding for preterm babies may be supported by enabling the baby to practice non-nutritive sucking before they are ready for oral feeding [12], having single family rooms to enable parents to stay with their babies 24 h a day [7, 13, 14], and a Family Integrated Care model, in which parents are supported to be their babies’ primary caregivers on the neonatal unit [15].

Recent guidance, quality improvement tools and accredited standards for neonatal units in the United Kingdom recommend giving parents early and consistent information about the value of breast milk; teaching mothers to express by hand and by pump; enabling mothers to express within two hours of birth and supporting them to express 8–10 times per 24 h in the first weeks; formally reviewing their expressing at least five times in the first two weeks and informally thereafter; promoting skin-to-skin contact; supporting mothers with positioning, attachment and recognising feeding cues; and giving mothers access to specialist support when needed [16–19]. This guidance positions these staff actions within the context of seeing parents as equal partners in their babies’ care; providing suitable facilities and equipment for expressing and for parents to be with their babies; training for all staff; and supporting feeding as a multidisciplinary team issue but with a dedicated lead professional.

The ability of neonatal units to implement these measures has been hampered by chronic and serious understaffing [20], with only 71.1% of neonatal nurse shifts staffed to recommended levels in 2022 [21]. There are also wide disparities in breast milk feeding rates between individual neonatal units measured by the proportion of very preterm babies receiving some of their own mother’s milk at the time of discharge from neonatal care. Nationally, 60.1% of very preterm babies received their own mother’s milk at discharge in 2022, either exclusively or combined with another form of feeding, but rates in local networks of neonatal units ranged from 48.6 to 79.3% [21, 22]. These disparities are likely to reflect service organisation and delivery issues within individual neonatal units as well as local background population rates of breastfeeding, which are affected by socio-demographic factors [20, 23–25].

Staff on neonatal units have an essential role in providing mothers of preterm babies with information and support to establish and maintain breast milk feeding and breastfeeding [26]. There is, however, very little evidence about health professionals’ experiences of supporting mothers of very preterm babies with breast milk feeding and breastfeeding, and how this may contribute to disparities between units. The 2019 report from the National Neonatal Audit Programme [27] recommended that neonatal units in England and Wales should identify barriers to breastfeeding across the patient pathway. The aim of our study was to explore and compare health professionals’ experiences of barriers and facilitators to their own role in supporting breast milk feeding and breastfeeding across the patient pathway in neonatal units in England. This study is part of a programme of work which also explored the experiences of mothers of very preterm babies at the same neonatal units [28].

## Methods

This was a qualitative comparative interview-based study, theoretically informed by phenomenological social psychology, which focuses attention on participants’ lived experiences and the meanings of social interactions [29]. It had a descriptive design, so aimed to stay close to participants’ accounts of their experiences while acknowledging the role of both participants’ understandings and the researchers’ interpretations in the production of knowledge [30].

Four neonatal units in England were purposively selected based on their rates of breast milk feeding for very preterm babies at discharge [31]. Two units (called Units A and B in this article) were chosen because they had high rates of breast milk feeding at discharge compared with the national average and two because they had low rates (Units C and D). Units B and D were Neonatal Intensive Care Units providing tertiary level care for babies of all gestations and prolonged intensive care; units A and C were Local Neonatal Units providing initial care for babies down to 27–28 gestational weeks and short periods of intensive care. The target recruitment was three members of staff from each unit: an infant feeding specialist (‘specialist’ is used in this article to imply specialist responsibility rather than professional qualification), and two other members of staff from any professional background who had experience of working with very preterm babies and their families. The sample size was calculated on the basis that three staff per unit, interviewed in-depth about their professional experience of a specific aspect of their work, would provide sufficient information power [32]; this was reviewed during data collection and no additional recruitment was necessary. A key contact at each neonatal unit invited eligible staff to participate in interviews, and passed the contact details of those who agreed to the research team. Participant information and consent forms were emailed to participants at least 24 h in advance; informed consent was obtained at the beginning of the interview. Each participant took part in a single semi-structured telephone interview between March and November 2022. Interview topic guides were developed with the support of a Parent, Patient and Public Involvement (PPPI) group comprising representatives of charities working with families of very preterm babies.

Interviews were audio-recorded and professionally transcribed. Interview transcripts were analysed using comparative thematic analysis [33], in parallel with ongoing data collection. Interviews from each unit were initially treated as separate datasets. Transcripts were checked against audio-recordings and reread for familiarity. Coding was both deductive, guided by expectations derived from the literature and the PPPI group’s experience (for example ‘unit culture and leadership’), and inductive, responding to new points raised by interviewees (for example, ‘disconnect between postnatal ward and neonatal unit’). Codes were recorded using NVIVO software and were refined and combined as data collection continued. A comparative analytic stage was added in which the differences and similarities between the participating neonatal units were identified by comparing the coding derived from the four datasets, and sub-themes and themes were developed. JM analysed all transcripts and FA and AA each analysed a subset; codes and themes were discussed and agreed. The researchers had no prior relationship with interviewees, and reflected critically on their own varied personal experiences of infant feeding and professional experiences of working in neonatal units caring for very preterm babies and their parents.

## Results

### Participants

Twelve health professionals took part in interviews – three from each neonatal unit. Infant feeding specialists at each unit have not been separately identified to protect confidentiality. Interviews lasted 35–64 min (mean 45 min). Participants’ pseudonyms and occupations are shown in Table 1.

**Table 1:** Participating units and interviewees

Unit pseudonym	Level of neonatal care	Rate of very preterm babies receiving breast milk at discharge, compared to England average*	Rate of breastfeeding initiation in local population, compared to England average**	Interviewee pseudonym	Occupation
Unit A	Local Neonatal Unit	Higher rate	Above average	A01	Nurse
				A02	Allied health professional
				A03	Doctor
Unit B	Neonatal Intensive Care Unit	Higher rate	Average	B01	Nurse
				B02	Nurse
				B03	Nurse
Unit C	Local Neonatal Unit	Lower rate	Below average	C01	Nurse
				C02	Nurse
				C03	Midwife
Unit D	Neonatal Intensive Care Unit	Lower rate	Average	D01	Nurse
				D02	Nurse
				D03	Doctor

* Based on figures from the National Neonatal Audit Programme [31]

**Based on the last published figures for breastfeeding initiation by NHS Trust [34]

### Findings

**Fig. 1:**
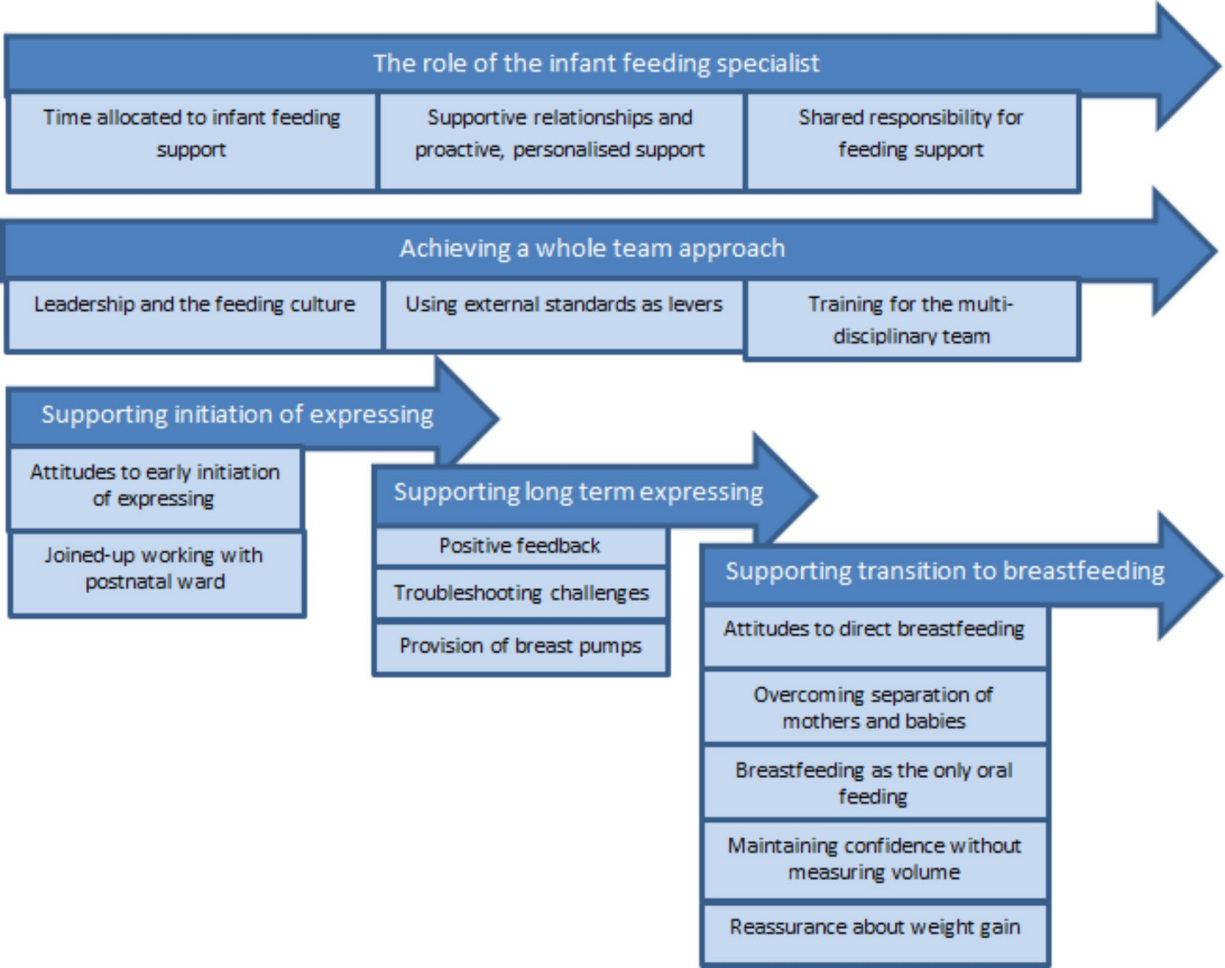
Themes and subthemes

Five topic-based themes were developed to describe the differences between the neonatal units in how they supported breast milk feeding and breastfeeding for very preterm babies, shown with subthemes in Fig. 1. The first two relate to the organisation of the neonatal unit (‘The role of the infant feeding specialist’ and ‘Achieving a whole team approach to breast milk feeding’), and the remaining three relate to key points in the feeding journey (‘Supporting initiation of expressing’, ‘Supporting long-term expressing’ and ‘Supporting the transition to breastfeeding’.)

### The role of the infant feeding specialist

#### Time allocated to infant feeding support

All the units had some staff who were designated as infant feeding specialists or leads, but the organisation of these roles varied greatly. At Unit A, the infant feeding lead had 15 h a week for this role, and had only basic training. At Unit B, there was a well-developed infant feeding team including a supernumerary full-time nurse dedicated to infant feeding support. Staff felt this had made an enormous difference to mothers.

*“Her role is purely to support mums who are expressing*,* breastfeeding and bottle feeding…We’ve definitely seen an increase in mums who are expressing*,* but also who are expressing for a lot longer.”* B02.

At Unit C, specialist support was provided by infant feeding midwives, but they were not encouraged to spend time on the neonatal unit except to support mothers who were under the care of the midwifery team, and their time could be reallocated to fill maternity shortages:

*“We do visit the neonatal unit if we’re asked to*,* however I’m paid by maternity services*,* so it’s coming out of the maternity budget not the neonate budget … I can be taken off to go and do clinical … and for that day there’s no [infant feeding support].”* C03.

At Unit D there was also very little staff time dedicated to feeding support, with the infant feeding nurse having only 7.5 h a week for her role.

*“We all have other jobs to do. And whilst I would absolutely adore to go and help mum straight after delivery and say*,* ‘This is your golden hour … this is when to start asking your boobs to start making milk*,*’ I’m actually usually ventilating the baby and putting lines in*,* so I can’t do both.”* D03.

At Units C and D, staff also highlighted that there was no consistent way to ensure that mothers who needed support were seen by the specialist.

*“If I found a mum who was really struggling with it*,* at that point we can ask an infant feeding sister to see that mum*,* but we don’t have a specific referral process for that*,* that would just be me off my own back trying to find the infant feeding sister.”* D02.

#### Supportive relationships and proactive, personalised support

Interviewees reflected on the ways in which an infant feeding specialist role could help mothers. Staff in Units A and B highlighted the benefits of being able to build a relationship over time, and taking responsibility for personalised continuity of care through the different stages of a mother’s feeding journey.

*“What we’ve changed massively is having that person who takes ownership to support these mums …I make a plan with my mums. I will know where each mum is at in her journey … If that mum needs me for an hour*,* I can sit with that mum for an hour.”* B03.

Another valued aspect of specialist roles at all units was that these staff proactively talked to mothers about how their expressing or breastfeeding was going, which was not necessarily something for which mothers felt able to seek help from busy nurses who were focused on clinical tasks. Infant feeding specialists were able to have these conversations sensitively and in the context of wider relational support that was not solely focused on maximising milk volumes for the baby, but was also empathetic towards the mother as an individual.

*“Being around to check in on them and see how they’re doing and explaining things to them. A lot of these mums tend to talk to me about how they’re feeling in general because I’m a separate person to who’s looking after their baby at that time.”* A01.

#### Shared responsibility for feeding support

Staff said that the effectiveness of a specialised role was maximised if the whole staff team saw support for breast milk feeding as a shared responsibility, with all staff understanding the importance of giving time-critical information and identifying mothers in need of additional help. However, staff at Units A, C and D reported that overstretched neonatal staff tended to over-rely on an under-resourced specialist who had limited availability, instead of incorporating basic feeding support into their own work. Consequently mothers could fall through the gaps.

*“Everybody pushes the responsibility of that particular role onto whoever else can do it. For instance*,* a woman has delivered the day before … she hasn’t had the conversation [about early initiation]*,* she’s had nothing until an infant feeding midwife has come in the next day.”* C03.

It was also noted that mothers’ confidence could be inadvertently undermined if they were asked about volumes of milk by lots of members of staff, insensitively and without co-ordination.

*“People have gone*,* ‘How much milk are you getting every time you express? Oh*,* that’s not enough you need to be getting this amount.’ … Some mums do then start to question whether they’re doing enough. We’ve had that feedback before*,* that asking too much pushes them away.”* D01.

To help make feeding support a whole-team responsibility while lactation was being established, Unit A had introduced a structured assessment tool to ensure that mothers were consistently asked about expressing by whichever nurses were on shift, every day for the first week. Interviewees at Unit D believed that a similar approach would help their team, but this had been opposed by colleagues because of a perception that this would add to their workload.

*“I tried to implement [an assessment checklist from Baby Friendly Initiative] but got told*,* ‘No*,* we don’t need more paperwork.’”* D01.

Shared responsibility for feeding support without adequate training also created problems when staff gave mothers non-evidence based or inconsistent information. Although interviewees at all four units reported that some staff gave mothers conflicting information, it was a particular problem at Units C and D.

*“The information that parents get is quite different from person to person … [Staff] give information based off their own experience or what they’ve seen*,* not necessarily with a mum who’s got a baby that’s 25 weeks.”* D02.

### Achieving a whole team approach to breast milk feeding

#### Leadership and the feeding culture

Interviewees highlighted the importance of leadership in supporting cultural change around breast milk feeding on neonatal units. In particular, leadership was needed to change staff perspectives, for example moving beyond seeing breast milk only as food to seeing feeding as part of a relationship between mother and baby. At Units A and B there were active efforts to persuade staff to see support for parents as integral to the neonatal nursing role, and to promote full partnership between parents and staff (Family Integrated Care).

*“The family needs to be seen as the baby with parents*,* [but] a lot of staff still like that little bit of control….As [an infant feeding] group we’ve got a very supportive matron. So it’s very much a team effort.”* B02.

In units where this leadership was not yet in place, staff who championed breast milk feeding described efforts to bring together a multi-disciplinary group and in particular to encourage buy-in from doctors and other senior staff:

*“Getting the right people on board*,* I think the higher you go*,* the better…That’s usually how it goes*,* if you can get doctors or paediatricians on board then you can get changes.”* C03.

#### Using external standards as levers

Unit B was actively working towards full Baby Friendly Initiative (BFI) accreditation [16], and Unit D had registered to begin the process. Interviewees at all units believed that BFI could be an important lever to drive change within their units.

*“BFI gives us a lot of access to educational tools and things that will help us*,* and I think it focuses the unit’s culture in terms of ‘watch our mouths’ when it comes to the language we use around breastfeeding and breast milk.”* D03.

At Unit C which was not engaged with BFI, an interviewee was trying to convince her colleagues to prioritise breast milk using other external standards, such as the PERIPrem bundle which includes promotion of maternal early breast milk [35] and the Saving Babies’ Lives care bundle for reducing perinatal mortality [36].

*“PERIPrem as a strategy*,* you have that as a leverage . To say [to colleagues]*,* ‘This isn’t about food*,* this is gut treatment*,* this is like giving the probiotics and the antibiotics.’”* C03.

At Unit A, interviewees noted that external criteria such as those used by the National Neonatal Audit Programme were influential but could have unintended consequences when staff prioritised supporting breast milk feeding (which was recorded and reported) but not direct breastfeeding (which was not).

*“We scored really high on all babies having breast milk*,* but in terms of babies actually being breastfed*,* we didn’t do that well. I think because it’s not something that’s particularly measured*,* there’s no funding attached to that*,* people are not maybe as vigilant or as committed to making it work.”* A03.

#### Training for the multi-disciplinary team

At Unit B, neonatal infant feeding training for all staff was a priority. At the other units, lack of training on expressing and breastfeeding specifically for very preterm babies was repeatedly mentioned as an obstacle to supporting mothers effectively.

*“The long term expressing is so much more challenging … The training that’s provided by the midwives is not really helping in that sense for us nursing staff … They do a small portion on preterm babies but most of it’s about full-term babies.”* A01.

This specific training was felt to be important for doctors as well as nurses, as some parents turned for advice to professionals who they perceived as more senior, to double check information given by nurses with more training:

*“If [doctors] had the same training as [nurses]*,* that would be a real positive in terms of helping certain families who like that reassurance from somebody more senior.”* D02.

Interviewees suggested that infant feeding training should also include communication skills because parents, who were trying to make sense of a confusing and stressful situation, were liable to read unintended meanings into what staff said.

*“Parents pick up on so much*,* staff sometimes think that parents aren’t listening or aren’t aware*,* but they are*,* they’re full of adrenaline*,* they’re very acute …[It’s] a very frightening situation as well*,* very vulnerable … so I think body language is a massive thing. Use of words*,* definitely.”* B02.

Interviewees also acknowledged that training was not the whole solution, as it was not necessarily enough to change the practices of some colleagues who were set in their ways. This required deeper cultural change.

*“On our unit a lot of the staff have been there for a really long time and you can’t change attitudes that are ingrained in people …They’ve had the infant feeding coordinators come in to do specific training with them … so there’s no excuse really.”* A03.

### Supporting initiation of expressing

#### Attitudes to early initiation of expressing

Interviewees at all four units were keen to motivate mothers to express their colostrum and then breast milk, by explaining its unique benefits. They encouraged mothers to start expressing milk irrespective of how they planned to feed their babies later.

*“At the beginning my question to a mum isn’t*,* ‘How do you want to feed your baby?’ It’s*,* ‘Would you be willing to express your breast milk*,* because actually your milk is golden medicine for your baby?’ A 22-weeker*,* it doesn’t matter how a mum wants to feed down the line … it’s actually the importance of expressing that milk and getting that milk into the baby.”* D01.

They noted that this approach was often successful for mothers who had no intention of breastfeeding.

*“Once you explain to parents the importance of the breast milk*,* a lot of parents will change their mind … even if they don’t go on to breastfeed but just give the milk by bottle…‘We didn’t want to do this*,* but because my baby is poorly I’ll do it for a little while.’”* C02.

Interviewees at all the units believed that it was beneficial, where possible, to talk about feeding with mothers before the birth, as they might be too distressed to absorb this information postnatally. At Unit B, the infant feeding nurse spent time with all pregnant women admitted to the hospital with the risk of premature birth. She explained feeding choices and the support available, underlined the importance of starting to express shortly after birth, and began to build a relationship with the mother that would continue throughout their baby’s time on the neonatal unit.

*“I sit with them and very gently ask them how I can support them with their choices of feeding. I go through the [feeding] pack*,* and I’ll say to them*,* ‘I will see you on the unit’… The hardest thing is getting mums to hand express within that hour of babies being born*,* and so I go through the importance of it … and I ask them to ask the midwives to help them with the hand expressing very quickly after birth. I’m almost giving them the confidence to say to someone*,* ‘Please will you help me?’”* B03.

At Units A, C and D, interviewees said there was no capacity for neonatal staff to do this and no system to alert neonatal staff when pregnant women were admitted at risk of very preterm birth. They also noted that doctors who saw the mother in the antenatal or immediate postnatal period did not necessarily give them time-critical information about starting to express.

*“It’s a battle… [Doctors] appreciate what breast milk is for a premature baby*,* but then they feel like it’s not a priority to be telling mum straightaway.”* D01.

#### Joined-up working with the postnatal ward

At all four units there were challenges in the working relationships between the midwives caring for mothers on the postnatal ward, and staff caring for the babies on the neonatal unit. Interviewees believed that when midwives did not promote early expressing this could be due to lack of time, lack of understanding, or feeling protective of the wellbeing of the mother in the short term.

*“There is quite a lot of resistance from the midwives*,* quite often the parents aren’t shown how to express or encouraged to express on the wards … The midwives are very much*,* ‘The women’s their job*,* once the baby’s born it’s not their problem.’ … So*,* where we will say*,* ‘Have a go at expressing*,* it’s really important to get it started*,*’ then they go back up to the ward [and] they get the opposite.”* C02.

*“The midwives have obviously got a lot of other things going on and being able to sit with [mothers] in a dedicated supportive fashion and teach them a brand new skill*,* it’s time-consuming and sadly that time is not always freely available*,* gets interrupted*,* gets put off*,* or mum’s tired because she’s delivered and they go*,* ‘We don’t want to push her*,*’ and it gets delayed and delayed.”* D03.

At Unit C, there were also inconsistent policies between the neonatal unit and the postnatal ward on *how* best to initiate expressing after very preterm birth, leaving mothers confused in the middle.

*“We know that getting babies’ mums on the pump is beneficial right from the very beginning*,* and they’re still being told by our midwifery team that you can’t pump for 48 hours*,* you should just hand express … I had a mum where I said to her*,* ‘Yes*,* go and pump now.’ ‘But the midwife told me not to.’”* C02.

At Units A and B, the infant feeding specialist from the neonatal unit would visit mothers on the postnatal ward to proactively offer support with early initiation of expressing. They had to overcome initial opposition from postnatal staff and build trust to be ‘allowed’ to support mothers:

*“It was very much ‘them and us’ … To begin with when I went over to [the postnatal ward] they were a little bit*,* ‘Who is this? Why is she interfering?’ But now they know I’m a great help to them …I’m taking a job away from them which is another job that they’re sometimes struggling to get round to doing.”* B03.

### Supporting long term expressing

#### Positive feedback

Interviewees at all four units said that giving mothers praise and positive feedback was important at all stages to reinforce their motivation, but they particularly emphasised the challenges mothers faced in expressing for weeks or months before a very preterm baby was able to feed orally.

*“I think a constant reinforcement of the importance of her breast milk*,* and also the fact that we recognise mums*,* how hard it is and how laborious it must be*,* and how committed they are in order to provide breast milk for their baby for such a long period of time.”* B02.

However this promotion of breast milk had to be handled sensitively, as some mothers were unable to produce enough milk or found it too difficult to keep up the demanding schedule of expressing, particularly if they were looking after other children. Interviewees recognised that worrying about maintaining the milk supply could in itself reduce the milk supply, and some continued to emphasise the process rather than the amount of milk produced.

*“We try to take the pressure off*,* because it is a lot of pressure… The more they stress*,* the less they produce. … So we say*,* ‘Just do what you can and the baby will get fed anyway.’”* C01.

#### Troubleshooting challenges

At all the units, specialist staff responded to mothers’ milk supply difficulties by personalised troubleshooting with them to consider ways they could increase lactation, such as lifestyle changes (eating well and resting), spending time skin-to-skin with the baby, power pumping (a session of short periods of repetitive pumping interspersed with short rest periods, intended to mimic a baby cluster feeding [37]), pumping regularly including at night, and taking medication. When a mother was highly motivated but her milk supply remained insufficient to meet her baby’s needs, specialist staff tried to support the mother to avoid feeling she had failed her baby, by helping her to understand possible reasons and to focus on what she had achieved.

*“It may help them come to terms with the fact that what they’re doing is absolutely fantastic*,* but we’ve perhaps got to the limit of what they’re going to be able to do. …Going through their history*,* going through what they’ve done since they started to express*,* they can then visualise*,* ‘Actually*,* I’ve done everything you’ve asked me to do and obviously my body is not going to be able to do anything more.’”* B02.

#### Provision of breast pumps and facilities

There were differences between units in the equipment provided to support expressing milk. Unit D had fewer pumps than cots so mothers had to queue to express milk if the pumps were in use:

*“We don’t have enough pumps on the unit*,* ideally it’s a pump per space*,* isn’t it? That would be great. But actually*,* let’s go for a pump in each bay and then have the expressing room kitted out with pumps*.” D01.

Units B, C and D had hospital grade breast pumps to lend to mothers for expressing at home while their baby was on the unit, but Units C and D did not have robust systems for monitoring the return of these free loans, and at both sites most of the loaned pumps were currently missing.

All of the units had a separate room where mothers could express milk, but interviewees noted that these were not particularly comfortable places where mothers would feel relaxed, which would encourage milk flow. They also said that while they encouraged mothers to express beside their babies, this could be difficult if the unit had not been designed with this in mind, and the cots were in a cramped, crowded environment with little privacy.

*“I don’t think parents feel that they can sit and express cot-side very often … It’s a very noisy*,* stressful environment…We don’t have curtains*,* we only have screens on wheels*,* which aren’t the greatest and they’re not always available.”* B02.

### Supporting the transition to breastfeeding

#### Attitudes to direct breastfeeding

The units had different levels of success in supporting mothers to begin directly breastfeeding when their babies were developmentally ready, and these reflected different staff attitudes. Breastfeeding was strongly supported at Unit B, where there was intensive support from specialist staff to help mothers and babies with the transition. Mothers were supported to introduce babies to the breast at a much earlier stage than in the other units, which was believed to be motivating as well as enabling mothers and babies to learn the practical skills of breastfeeding over a much longer period.

*“If I’ve got a 28-weeker who is stable*,* I look at all the observations*,* I’ll also talk to the medical team … I think it’s so important that these babies start tasting*,* and the closeness as well for mum. All that hard work these mums have done to get to this point*,* so they feel that they’re getting somewhere.”* B03.

At Unit A, breast milk feeding was a high priority for the wider staff team but encouraging direct breastfeeding was not, as noted above under subtheme ‘Using external standards as levers’. Likewise at Units C and D, establishing breastfeeding was not seen as particularly important or even feasible, compared to bottle feeding with expressed milk.

*“I don’t think we’ve recently had many mums go home exclusively breastfeeding and I don’t blame them to be honest*,* it’s just really hard … I think most mums are happy if it’s their milk to bottle feed anyway.”* C01.

#### Overcoming separation of mothers and babies

All of the units had transitional care rooms where parents could stay immediately before their baby was discharged, but none had space for all parents to routinely room-in. Staff at all four units said that having only limited facilities for parents to stay on site was particularly problematic at the point where the mother was trying to establish direct breastfeeding, and therefore needed to be with her baby as much as possible.

*“If we had more facilities where the mums could room-in and establish breastfeeding 24/7 they might be more successful with exclusive breastfeeding*,* but we’ve only got two parent bedrooms and the mum is only there at the time of discharge.”* C01.

At Unit A, night staff were reported to obstruct mothers’ attempts to spend time with their babies on the ward to breastfeed at night.

“*They don’t want mums on the unit at night*,* because one of them even said to the mum*,* ‘This is not a hotel*,* you can’t come and go as you please.’”* A03.

At Unit B, mothers were encouraged to room-in while they were establishing breastfeeding, but there could be competing needs for the limited rooms such as accommodating the parents of a critically ill baby. To avoid the problem of separation the unit had changed their discharge criteria so that mothers could establish breastfeeding at home with the support of an an outreach team:

*“As soon as baby starts to show feeding cues for breastfeeding … we will get that mum to come in and stay until their baby goes home… It used to be the fact that [to be discharged] babies had to be having half and half*,* so half their requirements would be either by the bottle or by the breast*,* and then the other half could be down the tube … Things have now changed where [with] our outreach team*,* as soon as the baby is having at least one breastfeed a day and we can see effective feeding and mum recognises effective feeding*,* then we get that baby home for her then to establish her breastfeeding at home.”* B02.

#### Breastfeeding as the only oral feeding

At all four units there was pressure from some staff for mothers to agree to give expressed milk or formula by bottle, rather than continuing partial tube feeds while the mother and baby established breastfeeding. Staff typically proposed this as a solution when breastfeeding was slow to be established, or because of their desire to respond to the baby’s feeding cues when the mother was not on the unit.

*“If a baby’s awake and rooting for a feed*,* we ask the mum would she consent to a bottle feed*,* because it’s not nice tube feeding a baby that’s awake and wanting a feed.”* C01.

Pressure to introduce bottles could also be driven by a lack of time or skills to support the transition to breastfeeding, which could be a long process for a very preterm baby.

*“Because of our short staffing the priority of most people is to do those things like basically keeping the babies alive*,* and although breastfeeding is an addition helping them thrive*,* it’s not in that moment going to keep them alive.”* D02.

This pressure could undermine mothers’ resolve to breastfeed, and some staff were also reported to make unhelpful critical comments to mothers who were trying to establish breastfeeding.

*“It’s a difficult culture to change because the staff are quite fixated on ‘The baby needs to bottle feed and if the baby’s looking to suck*,* why are we not offering bottles’?’ … [They] make the mum feel really bad about wanting to breastfeed or that she’s not there in the evenings*,* so it makes it very difficult and very uncomfortable*,* then all the work that you have done before gets undone.”* A02.

#### Maintaining confidence without measuring volume

Parents sometimes preferred to introduce bottles of expressed milk or formula rather than establish breastfeeding, because there was an objective measure of the volume of milk a baby had taken. Interviewees said they tried to reassure parents about other ways of assessing the efficacy of a breastfeed, but this was time-consuming.

*“Parents get very institutionalised with focusing on numbers*,* so if they can see that the baby’s had a certain number [of ml] then they feel less anxious*,* whereas a breastfeed you’re not seeing any numbers …You’ve got to find other things to make them feel confident in what they’re doing. It’s time again*,* just spending time with them.”* B01.

Less experienced staff might undermine mothers’ confidence by communicating their own desire for certainty:

*“Some staff members*,* they’re more interested in the figures*,* how many mls a baby has had. With more experience you know whether a baby has [breast]fed properly or not.” C01*.

Once a mother had begun direct breastfeeding, nurses typically used a time-based rating scale to assess the efficacy of a feed and thus the amount of top-up that would be given by tube. However, some interviewees said that that these mechanistic ways of assessing breast milk transfer were flawed, and again the key support was to build a mother’s own confidence to assess a feed by observing the baby and her own bodily sensations.

*“There’s a general expectation that there’s some kind of linear relationship between the length of a feed and how much milk has been taken in*,* and as many times as I tell them that’s complete nonsense*,* [the nurses] still ask me. Because they want a plan … I talk to the parents about how their breasts are feeling before*,* during and after a feed*,* what the babies look like when they’re feeding*,* are they actively feeding or are they having a few sucks and then drifting off to sleep?”* D03.

#### Reassurance about weight gain

All the interviewees were aware that it was common for babies’ weight gain to falter briefly when they began to fully breastfeed. They tried to reassure parents that temporary slowing of growth was usual, but they described interactions where colleagues had undermined mothers’ confidence by telling them that that this was a sign that breastfeeding was not succeeding, so top-ups should be given.

*“Sometimes the difficulty has been where mum has mostly established breastfeeding*,* and the baby has maybe dropped 50 g*,* and then [staff say]*,* ‘Oh well*,* you need to top up with a bottle or you need top up with a tube.’ And then it’s all panic stations.”* A03.

On Unit B, fully breastfed babies could be given a ‘shot’ of breast milk fortifier just before a breastfeed to help maintain their growth. As described above, this unit had an early discharge policy that did not require weight gain before discharge but enabled mothers to initiate breastfeeding on the unit, and their babies to be discharged primarily tube feeding, so that breastfeeding could be established at home with the support of an outreach team.

## Discussion

Comparing the experiences of staff at four neonatal units in England has revealed substantial variation in support for breast milk feeding for very preterm babies across different time points in the neonatal feeding journey from pre-admission to discharge, despite the same guidance, quality improvement tools and accredited standards being available to all units [16–19]. Unit B had a higher rate of breast milk feeding at discharge compared with other neonatal units in England, despite an average breastfeeding initiation rate in the local population. It had committed leadership, a dedicated full time infant feeding specialist who saw mothers on the antenatal and postnatal wards as well as the neonatal unit, regular infant feeding training for all staff, and a culture of Family Integrated Care. Unit A, which had a higher rate of breast milk feeding at discharge that may have been influenced by an above average rate of breastfeeding initiation in its local population, had an infant feeding specialist with 15 h a week of protected time. Staff at Unit A said that their high rate of breast milk feeding at discharge masked a low rate of direct breastfeeding, which was not an indicator measured by the National Neonatal Audit Programme, although it is recorded in neonatal electronic healthcare records.

Units C and D had a lower rate of breast milk feeding at discharge and an average (Unit D) or below average (Unit C) breastfeeding initiation rate in the local population. There was minimal protected time allocated to the infant feeding specialist role (Unit D) or reliance on infant feeding midwives from the postnatal ward (Unit C). Despite the commitment of individual members of staff, there was no strategic leadership or systematic support for breast milk feeding and breastfeeding across these units, and some staff were fatalistic in their perception that exclusive direct breastfeeding at discharge was an almost unattainable goal for a very preterm baby. These issues resulted in missed opportunities to support mothers effectively at each point on the feeding timeline, echoing the findings of Shattnawi [38] that overworked neonatal staff saw breastfeeding promotion as ‘a nicety instead of a necessity’ and abdicated the responsibility to support mothers to others.

To achieve cultural change, Bliss recommends that each neonatal unit should have a dedicated lead professional to champion the issue and support mothers, and also that feeding should be seen as an issue for the multidisciplinary team, who should be trained on the benefits of breast milk, lactation physiology, and how to support parents [19]. The experience of these four neonatal units illustrates the importance of these two recommendations being implemented together. Where the lead professional role existed but was under-resourced, inadequately trained staff would nonetheless treat support for breast milk feeding and breastfeeding as the responsibility of the lead, whether or not the lead was present on the ward. The support and information that staff gave parents was uncoordinated and inconsistent, and the time-critical optimal window for initiating lactation could be missed. By contrast, an adequately-resourced infant feeding lead could build an empathetic relationship with mothers that enabled her to give proactive, personalised, and consistent feeding support throughout the very preterm babies’ time on the neonatal unit. Where this role was part of a core infant feeding team and embedded in a multi-disciplinary staff team who regularly took part in infant feeding training that was specifically relevant to preterm babies, mothers could be effectively supported to initiate and sustain prolonged expressing and then to transfer to direct breastfeeding, which is associated with longer term breast milk feeding after leaving the neonatal unit [7, 10]. The importance of protected time for the infant feeding lead role has been recognised in guidance [18], but current guidance does not specify specialist staffing levels [16], leading to extreme variation in the allocated hours, banding and job descriptions between neonatal units in England (Personal communication from Karen Read, Neonatal Professional Lead for UNICEF Baby Friendly Initiative, shared with permission).

The policy focus in England on improving information sharing and partnership working between midwives and health visitors in the community [39, 40] has not extended to a similar focus on partnership between midwifery and neonatal care. This study has highlighted the gaps for mothers antenatally if the neonatal team were not aware of admissions of mothers at risk of very preterm birth, and postnatally if there was no joined-up working between midwives caring for the mother on the postnatal ward and the neonatal team caring for the baby, contrary to guidance from the Baby Friendly Initiative (BFI) [16]. In some cases mothers were given directly contradictory advice by nurses and midwives about when or how to start expressing, or midwives were actively opposed to neonatal infant feeding staff supporting ‘their’ mothers on the postnatal ward. Some neonatal units in Sweden and Finland offer ‘couplet care’, where mothers stay on the neonatal ward from birth and receive postnatal care for their own recovery there [41]. In this model it is the postnatal team which moves around the hospital, rather than the mother, or there may even be an integrated team with midwives employed as part of the neonatal team and extensive cross-training between the nurses and midwives [42]. While most English neonatal units lack the space and resources to reorganise care this way, neonatal and maternity staff could commit to joint working to provide integrated care for the mother-baby dyad, recognising both the mother’s own need to rest and recover and the importance of the mother starting to express milk within the first hours after very preterm birth in order to optimise her chance of reaching and maintaining a full supply [16, 17].

Previous research on training neonatal nurses in breastfeeding support for preterm babies found that this significantly increased the rate of preterm babies who were exclusively breastfed at discharge [26]. This study indicates the importance of extending training to neonatal doctors and the whole multi-disciplinary team, and indeed to any health professionals involved in intrapartum care, fetal medicine and perinatal care, as recommended by the National Neonatal Audit Programme [22]. This can ensure that mothers who experience very preterm birth are given information about early initiation of expressing at the first appropriate opportunity by whichever health professional is caring for them; that they receive consistent information and support about sustaining expressing from any staff members they encounter; and that they are enabled to transfer to direct breastfeeding without being undermined by a focus on weight gain or inflexible discharge criteria. Training could also address the challenge for nurses of how to feed a baby who is showing sucking behaviour but whose breastfeeding mother is not able to be on the unit for all of the baby’s feeds.

It is well established that physical separation of parents and their baby in neonatal care is one of the causes of breastfeeding difficulties as well as delays in establishing the parenting relationship [4, 43]. Parents’ experiences are very different in units where they are able to stay with their babies all the time, either in a private single family room or with a cotside bed or reclining chair that they are encouraged to see as their own space [44]. Accommodating parents on the neonatal unit can facilitate Family Integrated Care where the family is seen as a team led by the needs and behaviour of the very preterm baby, and the role of staff is conceptualised as supporting and guiding the family team in their feeding journey [45]. It has, however, also been noted that sleeping next to their baby may compromise the quality of parents’ sleep because of staff activity and noise; therefore parents’ mental health may be better protected by being offered a sleep space that is near but not with their baby [46].

None of the units in this study had the space or facilities to routinely offer parents the ability to room-in with their babies until just before discharge. Staff said this both hampered the establishment of direct breastfeeding, and discouraged some mothers from even trying, because they believed their baby was likely to meet the discharge criteria about oral feeding and weight gain sooner if bottlefed, as also reported by Bonet et al. [47] for French and English neonatal units. Unit B had addressed this issue in two ways: firstly by encouraging mothers to start putting their babies to the breast much earlier so that the transition to breastfeeding was a gentle process of familiarisation and closeness long before there was any nutritive feeding; and secondly by changing their discharge criteria so that a baby could be discharged home with a nasogastric tube once breastfeeding was successfully initiated. This meant that mother and baby could transition to full breastfeeding at home without separation, with the active support of an outreach team from the neonatal unit. This system contrasts with the policies in Italy reported by Bonet et al. [47], where very preterm babies were bottlefed on the neonatal unit before being discharged to begin breastfeeding at home. There was as yet no evidence on the impact of Unit B’s approach on mothers’ ability to sustain longer term direct breastfeeding for very preterm babies, and given the lack of literature about the effectiveness of this practice [48], this could be a topic for future research.

Irrespective of facilities for rooming in, the BFI standards require that parents should have 24-hour access to their babies, consistent with valuing them as partners in care, with policies in other paediatric settings, and with the Convention on the Rights of the Child article 9.1 [49]. Although all four units adhered to this principle in theory, some night staff at Unit A were reported to create an unwelcoming environment for parents, whose presence they found annoying, similar to some staff observed by Shattnawi [38]. This underlines the importance of building a culture of support for feeding as part of Family Integrated Care throughout the whole neonatal team.

### Strengths and limitations

It was a strength of this study that it involved health professionals from a range of professional backgrounds, working in four different neonatal units of different types in England and with different local population breastfeeding rates, so giving insight into the successful and less successful implementation of national policy and practice recommendations. The interviewees included both staff with a lead responsibility for supporting feeding for very preterm babies and staff with general clinical responsibilities. It was a limitation that all the staff who volunteered to be interviewed had a positive personal attitude to breast milk feeding and breastfeeding, so information about staff with less positive attitudes relied on interviewees’ descriptions of colleagues. Transferability may be limited by the variety in organisation of neonatal care between different health systems.

## Conclusion

There are different challenges for health professionals in supporting mothers of very preterm babies at different stages of their feeding journey, but the variation in practice in this study shows that there is much to learn from neonatal units that have high rates of breast milk feeding at discharge. Effective support can be influenced by having a supernumerary post dedicated to infant feeding; strong leadership that champions breast milk feeding and breastfeeding within Family Integrated Care; maintaining accountability by using existing quality improvement tools and accredited standards for neonatal units; and training for the whole multi-disciplinary team that encourages and enables every member of staff to take an appropriate share of responsibility for consistently informing and assisting mothers when the specialist is not available. National guidance could define recommended staffing levels for infant feeding support. Even when neonatal units do not have rooms for parents to live with their preterm babies, unit policies can support the transition to direct breastfeeding by encouraging mothers to start the process at the earliest opportunity, and potentially minimise separation by giving mothers sufficient outreach support to establish breastfeeding at home. Joined-up working between staff on antenatal and postnatal wards and neonatal units can enable integrated feeding support for the mother-baby dyad, overcoming the risk of staff seeing the mother’s and baby’s needs as unconnected or opposed. The National Neonatal Audit Programme could consider reporting the rate of exclusive direct breastfeeding at discharge in addition to breast milk feeding, so that this transition is treated with equal importance.

## Data Availability

The datasets generated during the current study are not publicly available due to the consent process but are available from the corresponding author on reasonable request.

## References

[1] Belfort MB, Knight E, Chandarana S, Ikem E, Gould JF, Collins CT, et al. Associations of maternal milk feeding with neurodevelopmental outcomes at 7 years of age in former Preterm infants. JAMA Netw Open. 2022;5(7):e2221608. 35816314 10.1001/jamanetworkopen.2022.21608 PMC9280396

[2] Gephart SM, Hanson C, Wetzel CM, Fleiner M, Umberger E, Martin L, et al. NEC-zero recommendations from scoping review of evidence to prevent and foster timely recognition of necrotizing enterocolitis. Maternal Health Neonatology Perinatol. 2017;3:23. 10.1186/s40748-017-0062-0 PMC5733736 29270303

[3] Dimitroglou M, Iliodromiti Z, Christou E, Volaki P, Petropoulou C, Sokou R et al. Human breast milk: the Key Role in the maturation of Immune, gastrointestinal and central nervous systems: a narrative review. Diagnostics (Basel Switzerland). 2022;12(9). 10.3390/diagnostics12092208 PMC9498242 36140609

[4] Ikonen R, Paavilainen E, Kaunonen M. Preterm infants’ mothers’ experiences with milk expression and breastfeeding: an integrative review. Adv Neonatal Care. 2015;15(6):394–406. 26536173 10.1097/ANC.0000000000000232

[5] Liu C, Pan M, Lu X, Gao Y, Xu J, Chen X. Breastfeeding barriers for Preterm infants in neonatal intensive care unit environments: a systematic Assessment and Meta-Analysis. Breastfeed Med. 2024;19(7):505–14. 38666420 10.1089/bfm.2024.0041

[6] Lubbe W. Clinicians guide for cue-based transition to oral feeding in preterm infants: an easy-to-use clinical guide. J Eval Clin Pract. 2018;24(1):80–8. 28251754 10.1111/jep.12721 PMC5901413

[7] Maastrup R, Hansen BM, Kronborg H, Bojesen SN, Hallum K, Frandsen A, et al. Breastfeeding progression in preterm infants is influenced by factors in infants, mothers and clinical practice: the results of a national cohort study with high breastfeeding initiation rates. PLoS ONE. 2014;9(9):e108208. 25251690 10.1371/journal.pone.0108208 PMC4177123

[8] Parker LA, Sullivan S, Krueger C, Kelechi T, Mueller M. Effect of early breast milk expression on milk volume and timing of lactogenesis stage II among mothers of very low birth weight infants: a pilot study. J Perinatol. 2012;32(3):205–9. 21904296 10.1038/jp.2011.78

[9] Levene I, Quigley MA, Fewtrell M, O’Brien F. Does extremely early expression of colostrum after very preterm birth improve mother’s own milk quantity? A cohort study. Arch Dis Child Fetal Neonatal Ed. 2024;109(5):475–80. 10.1136/archdischild-2023-326784 PMC11347236 38442953

[10] Ericson J, Eriksson M, Hoddinott P, Hellström-Westas L, Flacking R. Breastfeeding and risk for ceasing in mothers of preterm infants-long-term follow-up. Matern Child Nutr. 2018;14(4):e12618. 29733102 10.1111/mcn.12618 PMC6175451

[11] Bonnet C, Blondel B, Piedvache A, Wilson E, Bonamy A-KE, Gortner L, et al. Low breastfeeding continuation to 6 months for very preterm infants: a European multiregional cohort study. Matern Child Nutr. 2019;15(1):e12657. 30136374 10.1111/mcn.12657 PMC7199087

[12] Foster JP, Psaila K, Patterson T. Non-nutritive sucking for increasing physiologic stability and nutrition in preterm infants. Cochrane Database Syst Rev. 2016;10(10):CD001071. 10.1002/14651858.CD001071.pub3 PMC6458048 27699765

[13] Wataker H, Meberg A, Nestaas E. Neonatal family care for 24 hours per day: effects on maternal confidence and breast-feeding. J Perinat Neonatal Nurs. 2012;26(4):336–42. 23111722 10.1097/JPN.0b013e31826d928b

[14] Domanico R, Davis DK, Coleman F, Davis BO. Documenting the NICU design dilemma: comparative patient progress in open-ward and single family room units. J Perinatol. 2011;31(4):281–8. 21072040 10.1038/jp.2010.120 PMC3070087

[15] O’Brien K, Robson K, Bracht M, Cruz M, Lui K, Alvaro R, et al. Effectiveness of Family Integrated Care in neonatal intensive care units on infant and parent outcomes: a multicentre, multinational, cluster-randomised controlled trial. Lancet Child Adolesc Health. 2018;2(4):245–54. 30169298 10.1016/S2352-4642(18)30039-7

[16] UNICEF UK. Guide to the UNICEF UK Baby Friendly Initiative Neonatal Standards. 2022.

[17] British Association of Perinatal Medicine. Optimising Early Maternal Breast Milk for Preterm Infants: A Quality Improvement Toolkit. London; 2020.

[18] British Association of Perinatal Medicine. Optimising maternal breast milk for Preterm infants Part 2. To discharge and beyond. A Quality Improvement Toolkit. London: British Association of Perinatal Medicine; 2022.

[19] Bliss. Bliss Baby Charter. 2020.

[20] Adams E, Harvey K, Sweeting M. Neonataology - workforce. GRIFT Programme National Speciality Report. NHS England; 2022.

[21] National Neonatal Audit Programme. Summary report on 2022 data. London: RCPCH; 2023.

[22] National Neonatal Audit Programme. National Neonatal Audit Programme (NNAP) 2022 data: extended analysis report. London: RCPCH; 2023.

[23] Bonet M, Blondel B, Agostino R, Combier E, Maier RF, Cuttini M, et al. Variations in breastfeeding rates for very preterm infants between regions and neonatal units in Europe: results from the MOSAIC cohort. Archives Disease Child - Fetal Neonatal Ed. 2011;96(6):F450. 10.1136/adc.2009.179564 20538713

[24] Oakley LL, Renfrew MJ, Kurinczuk JJ, Quigley MA. Factors associated with breastfeeding in England: an analysis by primary care trust. BMJ Open. 2013;3(6). 10.1136/bmjopen-2013-002765 PMC3693424 23794590

[25] Mitha A, Piedvache A, Glorieux I, Zeitlin J, Roué JM, Blondel B, et al. Unit policies and breast milk feeding at discharge of very preterm infants: the EPIPAGE-2 cohort study. Paediatr Perinat Epidemiol. 2019;33(1):59–69. 30698887 10.1111/ppe.12536

[26] Maastrup R, Rom AL, Walloee S, Sandfeld HB, Kronborg H. Improved exclusive breastfeeding rates in preterm infants after a neonatal nurse training program focusing on six breastfeeding-supportive clinical practices. PLoS ONE. 2021;16(2):e0245273. 33534831 10.1371/journal.pone.0245273 PMC7857627

[27] National Neonatal Audit Programme. 2019 annual report on 2018 data. RCPCH:London; 2019.

[28] McLeish J, Aloysius A, Gale C, Quigley MA, Kurinczuk JJ, Alderdice F. What supports mothers of very preterm babies to start and continue breast milk feeding neonatal units? A qualitative COM-B analysis of mothers’ experiences. BMC Pregnancy Childbirth. 2024;24(1):725. 39506695 10.1186/s12884-024-06910-4 PMC11542208

[29] Landridge D. Phenomenology and critical social psychology: directions and debates in theory and Research Soc. Personal Psychol Compass. 2008;2(3):1126–42.

[30] Pidgeon N, Henwood K. Using grounded theory in psychological research. In: Hayes N, editor. Doing qualitative analysis in psychology. Hove: Psychology; 1997.

[31] National Neonatal Audit Programme. 2019 report on 2018 data, data file. https://www.rcpch.ac.uk/resources/national-neonatal-audit-programme-transparency-open-data2019

[32] Malterud K, Siersma VD, Guassora AD. Sample size in qualitative interview studies: guided by Information Power. Qual Health Res. 2016;26(13):1753–60. 26613970 10.1177/1049732315617444

[33] Braun V, Clarke V. Using thematic analysis in psychology. Qualitative Res Psychol. 2006;3(2):77–101.

[34] NHS England. NHS England Statistical Release Breastfeeding Initiation, Quarter 4 2016/17. 2017.

[35] Health Innovation West of England. PERIPrem undated [ https://www.healthinnowest.net/our-work/transforming-services-and-systems/periprem/

[36] NHS England. Saving babies’ lives: version 3 2023 [ https://www.england.nhs.uk/long-read/saving-babies-lives-version-3/

[37] Kalathingal T, Manerkar S, Mondkar J, Kalamdani P, Patra S, Kaur S, et al. Comparison of two pumping strategies to improve exclusive breastfeeding at discharge in mothers of VLBW infants with low milk output - A pilot randomized controlled trial. Indian J Pediatr. 2024;91(9):906–12. 37794310 10.1007/s12098-023-04859-4

[38] Shattnawi KK. Healthcare Professionalsʼ attitudes and practices in supporting and promoting the breastfeeding of Preterm infants in NICUs. Adv Neonatal Care. 2017;17(5):390–9. 28787301 10.1097/ANC.0000000000000421

[39] Public Health England DoH. Health visiting and midwifery partnership – pregnancy and early weeks. 2015.

[40] Public Health England. Care continuity between midwifery and health visiting services: principles for practice. 2021.

[41] Westrup B. Family-centered developmentally supportive care: the Swedish example. Arch Pediatr. 2015;22(10):1086–91. 26382641 10.1016/j.arcped.2015.07.005

[42] Klemming S, Lilliesköld S, Arwehed S, Jonas W, Lehtonen L, Westrup B. Mother-newborn couplet care: nordic country experiences of organization, models and practice. J Perinatol. 2023;43(Suppl 1):17–25. 38086962 10.1038/s41372-023-01812-3 PMC10716037

[43] Li X, Li Y, Qian L, Han P, Feng H, Jiang H. Mothers’ experiences of breast milk expression during separation from their hospitalized infants: a systematic review of qualitative evidence. BMC Pregnancy Childbirth. 2024;24(1):124. 38341542 10.1186/s12884-024-06323-3 PMC10858471

[44] Flacking R, Dykes F. Being in a womb’ or ‘playing musical chairs’: the impact of place and space on infant feeding in NICUs. BMC Pregnancy Childbirth. 2013;13(1):179. 24053167 10.1186/1471-2393-13-179 PMC4015611

[45] Mörelius E, Sahlén Helmer C, Hellgren M, Alehagen S. Supporting Premature Infants’ Oral Feeding in the NICU-A Qualitative Study of Nurses’ Perspectives. Child (Basel Switzerland). 2021;9(1). 10.3390/children9010016 PMC8774582 35053641

[46] White RD, Consensus Committee on Recommended Design Standards for Advanced Neonatal C. Recommended standards for newborn ICU design, 9th edition. Journal of perinatology: official journal of the California Perinatal Association. 2020;40(Suppl 1):2–4. 10.1038/s41372-020-0766-2 32859957

[47] Bonet M, Forcella E, Blondel B, Draper ES, Agostino R, Cuttini M, et al. Approaches to supporting lactation and breastfeeding for very preterm infants in the NICU: a qualitative study in three European regions. BMJ Open. 2015;5(6):e006973. 26129632 10.1136/bmjopen-2014-006973 PMC4486942

[48] Collins CT, Makrides M, McPhee AJ. Early discharge with home support of gavage feeding for stable preterm infants who have not established full oral feeds. Cochrane Database Syst Rev. 2015;2015(7):Cd003743. 26154426 10.1002/14651858.CD003743.pub2 PMC7133780

[49] United Nations. Convention on the Rights of the Child. 1989.

